# Highly Constrained Intergenic *Drosophila* Ultraconserved Elements Are Candidate ncRNAs

**DOI:** 10.1093/gbe/evv011

**Published:** 2015-01-23

**Authors:** Andrew D. Kern, Daniel A. Barbash, Joshua Chang Mell, Daniel Hupalo, Amanda Jensen

**Affiliations:** ^1^Department of Genetics, Rutgers University; ^2^Department of Molecular Biology and Genetics, Cornell University; ^3^Department of Microbiology and Immunology, Drexel University College of Medicine; ^4^Department of Biology, Dartmouth College, Hanover, New Hampshire

**Keywords:** ncRNAs, ultraconserved elements, comparative genomics, natural selection

## Abstract

Eukaryotes contain short (∼80–200 bp) regions that have few or no substitutions among species that represent hundreds of millions of years of evolutionary divergence. These ultraconserved elements (UCEs) are candidates for containing essential functions, but their biological roles remain largely unknown. Here, we report the discovery and characterization of UCEs from 12 sequenced *Drosophila* species. We identified 98 elements ≥80 bp long with very high conservation across the *Drosophila* phylogeny. Population genetic analyses reveal that these UCEs are not present in mutational cold spots. Instead we infer that they experience a level of selective constraint almost 10-fold higher compared with missense mutations in protein-coding sequences, which is substantially higher than that observed previously for human UCEs. About one-half of these *Drosophila* UCEs overlap the transcribed portion of genes, with many of those that are within coding sequences likely to correspond to sites of ADAR-dependent RNA editing. For the remaining UCEs that are in nongenic regions, we find that many are potentially capable of forming RNA secondary structures. Among ten chosen for further analysis, we discovered that the majority are transcribed in multiple tissues of *Drosophila melanogaster*. We conclude that *Drosophila* species are rich with UCEs and that many of them may correspond to novel noncoding RNAs.

## Introduction

The comparative genomics revolution of the past decade rests upon the notion that variation in levels of sequence conservation along the genome are informative for defining functional genomic elements (e.g., Birney et al. 2007; Roy et al. 2010; Dunham et al. 2012). Functional regions (exons, enhancers, promoters, etc.) are predicted to be constrained by natural selection in their sequence evolution, and thus should show less sequence divergence between species than nonfunctional regions of the genome. Consistent with this expectation, sequence conservation information has substantially improved *ab initio* gene and RNA predication (e.g., Carter and Durbin 2006; Pedersen et al. 2006).

While sequence conservation is an appealing source of information, surprisingly little is known about the biological roles of many conserved sequences, particularly those that do not encode proteins. Human ultraconserved elements (UCEs) best epitomize this paradox. Bejerano et al. (2004) described hundreds of stretches of the human genome of length 200 bp or greater that are perfectly conserved in alignments of the human, mouse, and rat genomes, representing approximately 100 Myr of evolution. The vast majority of these elements occur in regions with no known annotation, and less than one-fourth of UCEs overlap a known transcript. Because their initial description, only limited progress has been made in elucidating the function of vertebrate UCEs. Some UCEs seem to serve a role in gene regulation (Bernstein et al. 2006; Lee et al. 2006; Pennacchio et al. 2006; Paparidis et al. 2007; Visel et al. 2008). Indeed some elements function specifically as distal enhancers for neighboring developmental genes (Pennacchio et al. 2006; Paparidis et al. 2007; Visel et al. 2008). This role in development is also supported by bioinformatic analyses which demonstrate clustering in regions enriched for transcription factors and developmental genes (Bejerano et al. 2004). Other elements have been shown to function as transcriptional regulators, a subset of which are altered in human cancer (Calin et al. 2007; Ferreira et al. 2012; Lin et al. 2012). However, knockout mouse strains of four separate UCEs showed no detectable effects on viability or fecundity (Ahituv et al. 2007). These results are particularly surprising given that each of these four elements had been previously shown to have tissue-specific in vivo enhancer activity in mouse transgenic assays (Pennacchio et al. 2006). Thus to what extent are UCEs essential for fitness and development of the organism?

Inferential evidence from population and evolutionary genetics suggests that UCEs are indeed very important for organismal fitness. UCEs are under strong purifying selection in human populations (Katzman et al. 2007), are depleted among segregating segmental duplications and copy number variants (Chiang et al. 2008), and are nearly indispensible within mammalian genomes over deeper evolutionary timescales (McLean and Bejerano 2008). An alternative hypothesis to explain the existence of UCEs is that they are simply mutational coldspots of the genome. Fortunately, we can test between these two hypotheses using predictions from probabilistic population genetic models. Such analyses demonstrate that human UCEs appear to be strongly constrained by selection and thus are predicted to be functional. Human UCEs were investigated using targeted resequencing from human populations and a hierarchical Bayesian analysis, and found to be under roughly 3-fold stronger negative selection (i.e., constraint) compared with nonsynonymous sites (amino acid changing sites; Katzman et al. 2007). Put another way, levels of selection on amino acid sequences, our previous gold standard for sequence conservation, are only a fraction of what we observe acting on UCEs in humans. This pattern also generalizes to the entire “tail” of the distribution of conserved sequences. For example, independent sets of conserved noncoding sequences (non-CDS), by varying definitions, are under strong selection in both humans (Drake et al. 2005) and *Drosophila* (Casillas et al. 2007).

Thus, while UCEs must be important to fitness, the question remains as to what aspects of fitness they encode. Here, we present a comprehensive set of UCEs within the *Drosophila* genome that we have uncovered using 12 fully sequenced fruit fly genomes. We show using population genetic data that these elements are highly constrained by natural selection both historically and currently within *Drosophila melanogaster* populations. Further we show that several UCEs are transcribed and thus likely correspond to novel ncRNAs.

## Materials and Methods

### Sequence Data Used

**Fig. 1.—:**
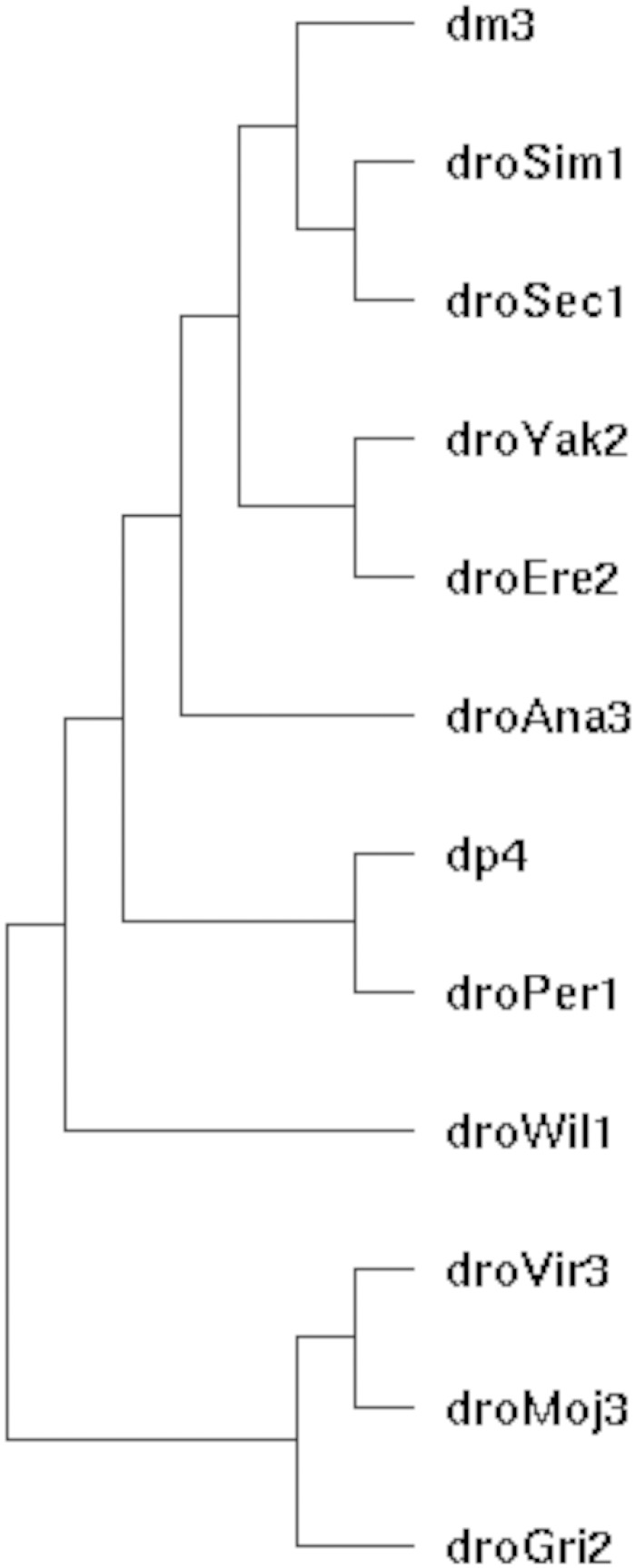
Phylogenetic tree of the species used for identification of UCEs. Shown are the assembly labels, see Materials and Methods for species names.

To search for UCEs specific to the genus *Drosophila*, we used the UCSC multiz alignment of 15 insect genomes pruned to exclude the three nondrosophilids. This includes the following assemblies that can be retrieved from the UCSC genome browser website (genome.ucsc.edu): *D*. *melanogaster* (dm3), *D*. *simulans* (droSim1), *D*. *sechelia* (droSec1), *D*. *yakuba* (droYak2), *D*. *erecta* (droEre2), *D*. *ananassae* (droAna3), *D*.*pseudoobscura* (dp4), *D*. *persimilis* (droPer1), *D*. *willistoni* (droWil1), *D*. *virilis* (droVir3), *D*. *mojavensis* (droMoj3), and *D*. *grimshawi* (droGri2). A phylogenetic tree of the species used is shown in figure 1. The majority of these sequence data was collected by Clark et al. (2007). All genic annotation is based on BDGP R5 data. modENCODE data were used for a subset of analyses. Population genetic variation data used were from the set of sequenced African *D*. *melanogaster* genomes produced an analyzed in Pool et al. (2012) as well as from a set of sequenced inbred lines derived from a North Carolina population (Mackay et al. 2012).

### Identifications of Elements

We searched the resulting alignment of 12 genomes using a simple program based on the UCSC genome browser source code (i.e., the kent tree) that records all ungapped, perfectly conserved sequences throughout a set of MAF blocks of some minimum length cutoff. Our program, mafUltras, is available upon request.

### Phylogenetics Methods

To determine the probability of observing a given UCE given a phylogenetic model we used the PHAST package (Hubisz et al. 2011). In particular, we estimated a phylogenetic model for all sites in our 12-way alignment using phyloFit (Siepel and Haussler 2004) and then calculated the probability of observing no substitutions in each of our element alignments, given the model using phyloP (Pollard et al. 2010), using the SPH method of Siepel et al. (2006). In addition we computed likelihood ratio tests (LRTs) of sequence evolution deceleration versus our null phylogenetic models using the LRT mode of phyloP. It is worth noting that each of these tests is conditional upon the length of each observed element. As the sequence composition and evolutionary properties of the heterochromatic and euchromatic portions of the genome are known to differ, we also estimated phylogenetic models separately for each of these genomic segments and used each for hypothesis tests as appropriate.

### Population Genetics Methods

To estimate selection coefficients in our UCEs, we used a previously published Markov chain Monte Carlo (MCMC) approach to estimate a genomic distribution of selection coefficients for new mutations that varies between site types (e.g., nonsynonymous, UCEs, etc.) while accounting for the divergence-based ascertainment (Katzman et al. 2007; Kern 2009). This method uses as input the derived allele frequency spectrum from sites of different classes and uses those data to estimate the mean and the variance of the distribution of selection coefficients for new mutations. Here, we considered three separate site types: polymorphisms in UCEs, regions flanking UCEs (1 kb in either direction), and nonsynonymous polymorphisms. The method has been used with success in both the Human and *Arabidopsis* genome (Katzman et al. 2007; Kritsas et al. 2012). For each data set we ran nine chains from overdispersed starting points of the parameters for 10^5^ iterations, which we sampled every ten iterations. The first half of each chain is treated as burn in and discarded; the second half is retained for estimation of the posterior distribution of parameters. Convergence was determined using Gelman’s multivariate potential scale reduction factor (Brooks and Gelman 1997). We summarize our Markov chains using maximum a posteriori (MAP) estimates of the parameters but we also give credible intervals for the posterior distributions to assess confidence in the point estimates.

### RNA Secondary Structure Analysis

We used the ViennaRNA package (Lorenz et al. 2011) to predict secondary structures associated with UCEs. In particular, minimum free energy structures were generated with the RNAFold program. To assess the significance of secondary structure predictions we used the method of Clote et al. (2005). Clote et al. (2005) suggest comparing the minimum free energy (MFE) of a sequence to the distribution of MFEs from exact dinucleotide randomizations of that sequence. These dinucleotide randomizations are generated via the Altschul–Erickson algorithm and then we folded them again using RNAFold. Finally, the standardized difference between the observed and expected MFE for a given sequence was expressed as a *Z-*score. We also considered predictions from the EvoFold program and its associated analyses of *Drosophila* alignments (Pedersen et al. 2006; Stark et al. 2007). The EvoFold program scans a multiple alignment of genomes for RNA structures using a probabilistic model called a phylogenetic stochastic context free grammar (phylo-SCFG). Evofold scores are log-likelihood ratios of two phylo-SCFG, an RNA model that allows for regions containing fRNAs and a background model that describes regions that do not contain fRNAs.

### RT-Polymerase Chain Reaction

To test whether a given UCE is transcribed we used RT-polymerase chain reaction (PCR) on RNA generated from embryos, larvae, and adult flies. Tissue samples were 0–4 h and 0–16 h embryos, 3- to 4-day-old larvae, and 3- to 5-day-old female and male flies (day 1 as the day of eclosion) from a *w^1118^ D*. *melanogaster* stock grown at 25 °C. Two biological replicates were made for each time point. Total RNA was isolated using Trizol reagent (Invitrogen), DNAseI (Roche) digested at 37 °C for 2 h, followed by 10 min incubation at 75 °C with the addition of ethylenediaminetetraacetic acid to 8 mM to inactivate the enzyme.

First strand cDNA synthesis was carried out using Superscript III kit (Invitrogen), following the manufacture’s protocol. Approximately 12.5 µg of DNAseI-treated RNA was used for each 50 µl reaction with either oligo(dT) or random hexamers as primers. Reverse transcription without reverse transcriptase (RT-control) was performed alongside of each cDNA synthesis reaction with random hexamers.

PCR reactions were carried out using GoTaq polymerase (Promega) in the following conditions: 95 °C for 30 s (first cycle 2 min), 55–65 °C (determined empirically for each primer pair) for 30 s, 72 °C for 10 s (last cycle 2 min), for 35 cycles. Primers used in PCR:chr3R.19 F/R: TTGCAACATCAAAATTTAACGAA/ATCGTGTCGCTCGTTTGTTT. chr3R.5 F/R: ACACTTCCTGTTTTTCTTATTCACTG/AATGGGTCATTTGCGTAT CC. chrX.3 F/R: CCTATTTATCCTGGCGTTGG/AAAAGTGCGCACAATTATTCA. chr3L.7 F/R: GGTTCGTGCGGCGTAATA/CGTACGTGCGCATATTTCAT. chr3R.10 F/R: TCCAAACTTAAGGAATTACTGAAAAA/TGTTTGAACTGATAATGTCCCAAG. chr3R.11 F/R: GTTGTCATGTACGAAAATTGTAGC/ \AAATTGATATGTTTGAACTATTTCCTG. chr3R.16 F/R: TTTTGCCTGATTTTGTGTGC / TCGAACAAATATGTTTACATTTAGCA. chr3R.17 F/R: CACCAACAACAGGAAGGAATG / CCAAAGTTGCACTCGACAAA

chr3R.2 F/R: TGCTCATGAATGATTTGTTGG/TGGAATTGCCCACATCAAAG chrX.6 F/R: CGCGATAAGGTAATTGGACTA/CTGCCGAAATGTCAAATGC. Primers for *Rox2* were from Meller et al. (2000); for *yar*, from Soshnev et al. (2011).

## Results

### Identification of UCEs

**Fig. 2.—:**
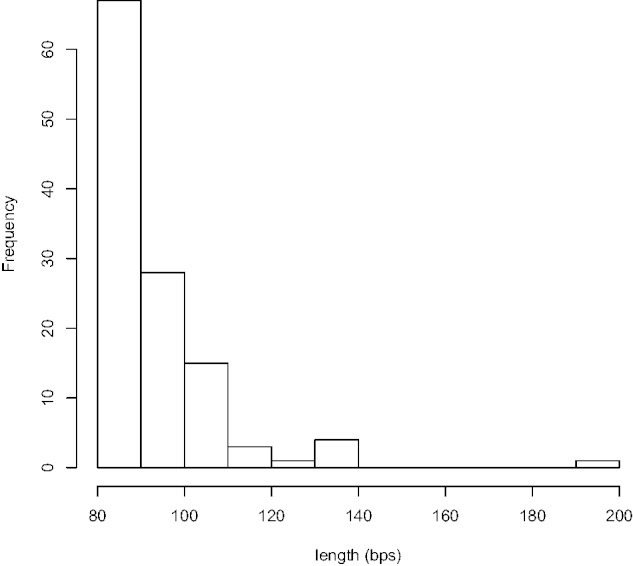
Length distribution of perfectly conserved sequences in the alignment of 12 *Drosophila* genomes of length ≥80 bp. By definition of an ultraconserved we find 98 elements in mapped regions of the genome, representing the top 0.1% of all conserved elements in length.

We searched multiple alignments of 12 *Drosophila* genomes (Clark et al. 2007) for completely conserved, ungapped regions. The *Drosophila* species used (fig. 1) represent approximately 50 Myr of evolutionary divergence since their most recent common ancestor, which is slightly less than that used in the original discovery of vertebrate UCEs (Bejerano et al. 2004). However, the total time in the *Drosophila* species tree is much greater than that used by Bejerano et al. (2004) because we included more species, so accordingly more information can be mined from the *Drosophila* comparison. An initial length cutoff of ≥50 bp found 1,306 conserved regions, thus establishing a liberal distribution of conserved region lengths. As we are interested in looking at the extreme tail of the distribution of conservation for genomic regions and biologically characterizing such a set of elements, we chose an arbitrary cutoff of ≥80 bp. This cutoff revealed a much more limited set of 119 genomic regions. Of these, 21 regions were from unmapped regions of the genome (chrU or chrUextra) and were not considered for further analysis. The length distribution of the remaining 98 elements is shown in figure 2. We found no UCEs >192 bp. UCEs are therefore significantly smaller in *Drosophila* than in vertebrate genomes. Perhaps this difference in size reflects the global difference in genome sizes as *Drosophila* genomes are roughly an order of magnitude smaller than mammalian genomes. However, it is less clear that smaller genomes lead to smaller functional elements. We could speculate that the known deletion bias of *Drosophila* (Petrov et al. 2000) when coupled with stronger natural selection in the large, outbreeding populations of flies may yield more streamlined genomes (e.g., Petrov 2001), and perhaps the regions of conservation might be reduced in length as a result.

Although our set of elements clearly represents the tail of the distribution of conservation, this is merely a statement about the empirical distribution and does not address what the probability of observing such conservation in our alignment would be. We therefore used the method of Siepel et al. (2006), which calculates the probability of observing a multiple alignment given an estimated phylogenetic model. Our background phylogenetic model was estimated both for the entire 12-way genomic alignment, as well as for the euchromatic portion of the genome alone, as all of the elements we have identified occur in euchromatin. For each of our UCEs, the probability of observing such perfect conservation throughout our multiple alignment given our estimated model was <1e-5 using either the SPH method (Siepel et al. 2006) or a log-LRT as implemented in the phyloP software (Pollard et al. 2010). This result is true for both the phylogenetic model estimated from the entire genomic alignment as well as that from the euchromatic portion alone. Thus the conservation we observe in these elements is highly significant.

### Population Genetics of UCEs

As stated above, two hypotheses are consistent with the extreme sequence conservation seen at UCEs: 1) mutational cold-spots or 2) strong negative selection on functional elements. Population genetic analyses of polymorphism data allow one to directly distinguish between these hypotheses as the two models (neutral vs. selected) predict different distributions of the frequency of new (derived) mutations. In particular, derived allele frequencies should be skewed toward being rarer under negative selection than they would under a neutral model with reduced mutation rate. This is exactly what was observed in humans (Katzman et al. 2007).

Using recent whole-genome sequence from a collection of 130 African *D*. *melanogaster* lines (Pool et al. 2012) and 154 North American *D*. *melanogaster* lines (Mackay et al. 2012), we estimated the distribution of selection coefficients (α = 2Ns) for new mutations at UCEs and compared the estimated mean of that distribution to the mean α from 1 kb of DNA sequence flanking the UCEs as well as to nonsynonymous variation from throughout the genome using the hierarchical Bayesian method of Katzman et al. (2007). Supplementary figures S1–S3, Supplementary Material online, demonstrate representative convergence of our chains to the posterior by ploting the posterior probability as it changes throughout iterations of our MCMC simulations for the African sample. Generally our simulations converged very quickly. We have summarized the posterior distributions of our estimated mean (μ) selection coefficients for each site type in table 1 for each population separately. Mean selection coefficients against new mutations at ultraconserved sites are roughly an order of magnitude stronger (Africa MAP estimate μ = −20.34; North America MAP estimate μ = −9.76) than the strength of selection against new missense mutations throughout the genome (Africa MAP estimate μ = −2.61; America MAP estimate μ = −0.68). Further this difference is not a function of differences in the genomic context of these mutations as mutations in sites immediately flanking UCEs also have weaker selection coefficients associated with new mutations in both populations (Africa MAP estimate μ = −1.9; North America MAP estimate μ = −0.68). The credible intervals of the posterior distributions of μ between ultraconserved sites and nonsynonymous sites are completely nonoverlapping, demonstrating a significant difference between these two classes. That the strengths of selection estimated from the North American population are weaker in magnitude is not surprising, given the demographic population history of out-of-Africa bottlenecks associated with the founding of the North American population (David and Capy 1988). However, rank orders of relative strength of selection across site types are maintained between populations, which lends strong support for the hypothesis that UCEs are under very strong constraint within populations. Indeed, the strength of selection we observe here is much stronger than for human ultraconserved regions (Katzman et al. 2007). We conclude that *Drosophila* UCEs are highly constrained both historically and currently and thus are highly likely to be functional.

**Table 1:** Population Genetic Estimates of the Mean Selection Coefficient (2Ns) of New Mutations at UCEs, sites flanking UCEs, and Nonsynonymous polymorphisms

Population	Site Type	SNP #^a^	2Ns		
			Posterior Mean	MAP	95% CI
Africa	UCE	137	−21.07	−20.34	(−27.12, −15.47)
	Flanking UCE	11,483	−1.90	−1.90	(−1.97, −1.82)
	Nonsynonymous	332,434	−2.61	−2.61	(−2.63, −2.59)
North America	UCE	83	−10.64	−9.76	(−13.14, −8.53)
	Flanking UCE	5,955	−0.18	−0.18	(−2.74, −0.07)
	Nonsynonymous	146,604	−0.68	−0.68	(−0.71, −0.66)

^a^Shown by column are the number of SNPs used for estimation and then three summaries of the posterior distribution from our MCMC simulations: the posterior mean, the MAP estimate, and the 95% credible set.

### Annotation of Drosophila UCEs

**Fig. 3.—:**
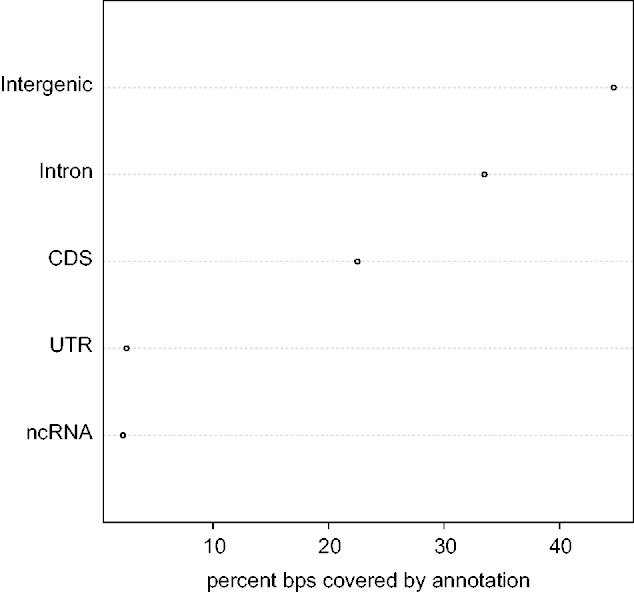
Ultraconverseved element coverage of annotation types throughout the *Drosophila* genome. Here, we show the percentage of base pairs within our UCEs coveraged by each annotation type in turn. Note that the single largest fraction of bases is covered by no known annotation yet cumulatively more bases are covered by portions of protein-coding genes (CDS, introns, and UTRs collectively) than not.

Where in the genome are UCEs found? The 98 mapped *Drosophila* elements include 9,152 bp or roughly 0.006% of the genome. Approximately 55.3% of these base pairs overlap some portion of a protein-coding locus (CDS, untranslated region [UTR], or intron; see fig. 3); however, only 22.5% overlap actual CDS. Approximately 33.5% of bases overlap known introns and a small percentage of bases (2.5%) overlap UTRs. Two elements overlap known RNA genes (2.2% of base pairs), both of which are snRNAs. This is in contrast to a previous set of insect UCEs based on three-way alignments (Glazov et al. 2005) that found some enrichment of UCEs in miRNA sequences. Finally, 44.7% of ultraconserved bases overlap no known annotation feature. This observation, that many UCEs are found in intergenic, noncoding DNA, mirrors what has been observed in the collection of vertebrate (Bejerano et al. 2004) and insect UCEs (Glazov et al. 2005).

### Genic UCEs

Fifty-two UCEs overlap some portion of a protein-coding locus (UTR, CDS, or intron; see table 2). These include genes crucial for early development in *Drosophila*, including the homeobox loci *Ubx, Antp,* and *hth*. Indeed a few loci harbor more than one UCE, such as *para* which contains five separate UCEs in its CDS, and *slo, hth,* and *Ubx* which each contain two elements.

**Table 2:** UCEs That Occurring in CDS

Chromosome	Start	End	Name	Symbol	Fbid
chr2L	2785538	2785672	chr2L.2	Syt1	FBgn0004242
chr2L	4314519	4314622	chr2L.4	tutl	FBgn0010473
chr2L	14089129	14089263	chr2L.5	nAcRalpha-34e	FBgn0028875
chr2R	10179256	10179343	chr2R.1	Ih	FBgn0263397
chr2R	20614061	20614184	chr2R.2	CG33988	FBgn0053988
chr3L	9148233	9148339	chr3L.1	Rdl	FBgn0004244
chr3L	12840178	12840256	chr3L.2	CG10948	FBgn0036317
chr3R	15589319	15589395	chr3R.2	GluClalpha	FBgn0024963
chr3R	20500491	20500603	chr3R.3	slo	FBgn0003429
chr3R	20508039	20508121	chr3R.4	slo	FBgn0003429
chr3R	27180238	27180336	chr3R.5	CG34347	FBgn0003429
chr3R	27663491	27663549	chr3R.6	RhoGAP100F	FBgn0039883
chrX	3678660	3678739	chrX.1	tlk	FBgn0086899
chrX	5293623	5293703	chrX.2	SK	FBgn0029761
chrX	14893890	14893967	chrX.3	eag	FBgn0000535
chrX	16365287	16365370	chrX.4	para	FBgn0264255
chrX	16367614	16367806	chrX.5	para	FBgn0264255
chrX	16371485	16371604	chrX.6	para	FBgn0264255
chrX	16403608	16403737	chrX.7	para	FBgn0264255
chrX	16408137	16408217	chrX.8	para	FBgn0264255
chrX	17845447	17845546	chrX.9	Sh	FBgn0003380

To ask whether loci containing UCEs are enriched for specific biological functions we used the DAVID annotation tool (Dennis et al. 2003). This analysis returned four annotation clusters with an enrichment score greater than 2 (see supplementary table S1, Supplementary Material online). Cluster 1 included gene ontology (GO) terms such as developmental protein (*P*-value = 6.11e-06), DNA-binding (*P*-value = 3.07e-05), DNA-dependent regulation of transcription (GO:0006355; *P*-value = 6.91e-05), and sequence-specific DNA-binding (GO0043565; *P*-value = 7.79e-05). This cluster includes the early development and homeobox genes found in table 2. Cluster 2 includes large ion channel genes and is enriched for GO terms such as ion channel complex (GO:0034702; *P*-value = 2.22e-06), alternative splicing (*P*-value = 6.43e-08), and gated channel activity (GO:0022836; *P*-value = 1.39e-05). Genes within this cluster include *slo, Sh, tutl,* and *rdl* (see supplementary table S1, Supplementary Material online). Cluster 3 mirrors to a large extent the terms found in cluster 1, with enrichments for DNA-binding (*P*-value = 3.1e-05) and sequence-specific DNA-binding (GO0043565; *P*-value = 7.79e-05), but adds to these terms homeobox-related terms such as homeobox (*P*-value = 0.0079), blastoderm segmentation (GO:0007350; *P*-value = 0.0097), embryonic pattern specification (GO:0009880; *P*-value = 0.012), and segment specification (GO:00073979; *P*-value = 0.0132). Accordingly, well known Hox genes appear in this cluster including *Ubx, Antp, dpp,* and *hth*. Finally, cluster 4 is enriched for developmental terms such as developmental protein (*P*-value = 6.11e-06), transcription regulator activity (GO:0030528; *P*-value = 1.9e-04), imaginal disk development (GO:0007444; *P*-value = 8.57e-04), and leg disk development (GO:0035218; *P*-value = 9.9e-04), as well as a host of other morphogenesis terms (see supplementary table S1, Supplementary Material online). Overall it seems that UCEs that overlap portions of protein-coding genes tend to be involved in development, transcriptional regulation, and ion channels.

Considering only the 21 UCEs that overlap CDSs in whole or in part (supplementary table S2, Supplementary Material online), we find enrichment for GO terms involved in ion channels and behavior (supplementary table S3, Supplementary Material online). Interestingly, 11 of the 16 loci that contain these 21 UCEs undergo ADAR-dependent RNA editing (Hoopengardner et al. 2003; Rodriguez et al. 2012). ADAR editing requires specific mRNA secondary structure forming as a result of complementarity among regions of the mRNA sequence. We propose that this structural constraint is responsible for the extreme evolutionary conservation we observe in these genes.

### Noncoding UCEs

Forty-seven UCEs are completely contained within noncoding portions of the genome. At least three functional hypotheses exist for the biological roles of intergenic UCEs: 1) they are enhancers that regulate transcription of nearby or more distant genes, as has been shown for some of the vertebrate UCEs (Pennacchio et al. 2006), 2) they are structural chromosomal elements, such as nuclear matrix attachment regions or chromosomal-counting elements (Chiang et al. 2008), and 3) they may encode unannotated noncoding RNA genes (ncRNAs). Indeed the complete set of noncoding UCEs may be a mixture of all three types of elements.

The *Drosophila* genome is relatively well annotated with respect to regulatory elements among animal genomes, so examining our first hypothesis above is straightforward. Using the ORegAnno database (Griffith et al. 2008) we queried for UCEs that overlap known regulatory elements. Only two noncoding UCEs had any overlap with ORegAnno elements, and in each case the ORegAnno element was very large with respect to the UCE (∼5 kb). Thus it is difficult to conclude that enhancers or other regulatory DNA elements represent a significant fraction of the elements we have discovered.

Nevertheless, UCEs might show a distinct pattern of base composition that contains regulatory information. We asked if Drosophila UCEs show changes in A + T frequency as has been noted in mammalian UCEs (Chiang et al. 2008). In comparing 60 bp of flanking sequence to the central 60 bp of our elements we found dramatic differences in A + T frequency: flanking regions show a mean A + T frequency = 0.44 whereas mean core ultra A + T frequency = 0.66 (mean A + T frequency in the *Drosophila* genome is 0.575 by comparison). This is a highly significant difference using a Wilcoxon rank sum test for difference in medians (*W = *60.5; *P*-value < 2.2e-16). Thus core regions of UCEs show greater A + T frequency than the genome whereas directly flanking sequences show a dip in A + T frequency. It has been suggested that such abrupt changes in base composition correlate with changes in DNA methylation or nucleosome positioning in mammals (Chiang et al. 2008).

**Fig. 4.—:**
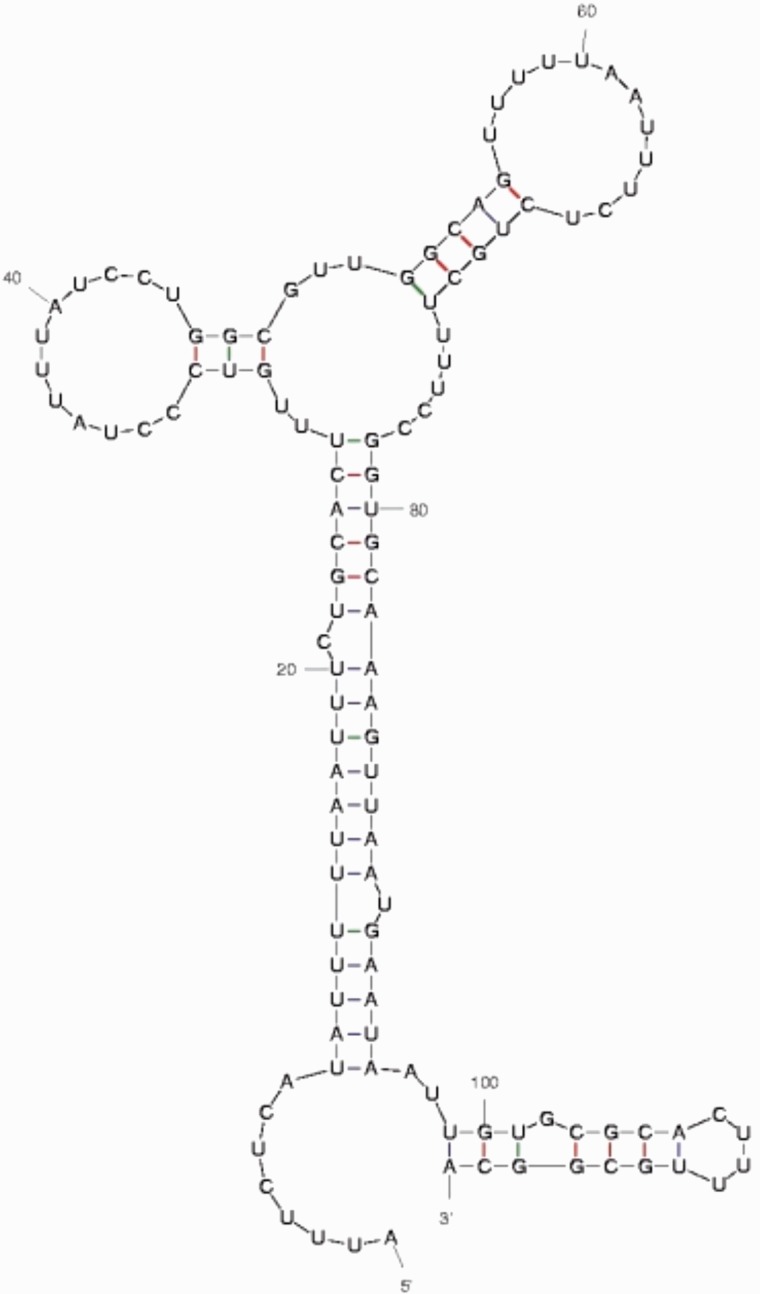
Predicted RNA secondary structure of the longest noncoding UCE X.3. This structure was predicted with the mfold algorithm and has a free energy ΔG = −18.71. We have subsequently confirmed transcription of this element (see fig. 5).

Preliminary examination of the longest noncoding UCEs suggests that many represent novel ncRNAs. The first piece of evidence comes from RNA secondary structure predictions using both comparative genomic approaches (i.e., phylo-SCFGs-Evofold algorithm; Pedersen et al. 2006; Stark et al. 2007), and single genome predictions (e.g., MFOLD algorithm; Zuker 2003). Forty-three of the 46 noncoding elements examined contain or are wholly composed of significant Evofold predictions from Stark et al. (2007). Strong Evofold predictions in this case are especially surprising, given that the algorithm uses information in multiple alignments about compensatory mutations in the RNA secondary structure. Because UCEs by our definition have undergone no substitutions, each of these structure predictions rests solely on the thermodynamic stability of the predicted molecules. We then examined MFEs associated with UCE secondary structures. In particular, we were interested in assessing if our observed MFEs were significant given the genomic background (See Materials and Methods). Secondary structures associated with our elements do indeed show a distribution of *z-*scores skewed toward negative numbers and thus significance (mean *z* = −0.309), though it is a not a very strong effect. Nevertheless, the observed average score is in-line with expectations from Clote et al. (2005) who showed that known structural RNA are only slightly biased toward negative *z-*scores. Notably, longer UCEs are biased toward more significant *z*-scores (Pearson’s *r = *−0.451, *P* = 0.0019) as we would expect if longer UCEs were more highly structured. Supplementary table S4, Supplementary Material online, provides a listing of each noncoding UCE analyzed for RNA structure along with associated MFE estimates, *z*-scores, and EvoFold scores. Figure 4 shows a secondary structure prediction of UCE X.3, the longest noncoding secondary structure that we found. This structure, with its three stem-loops radiating from a central spoke, is representative of many of the secondary structures we predict from these elements. Supplementary figure S4, Supplementary Material online, shows images of seven such predicted structures.

We examined these UCEs for evidence of transcription using a tiling DNA microarray study of transcription during early fly development (Manak et al. 2006), and RNA-Seq data recently generated as part of the modENCODE project (Roy et al. 2010). To our surprise only 15 of 46 intergenic UCEs show any evidence of transcription from these data, and in most cases the number of RNA-Seq reads covering an element was extremely low.

**Fig. 5.—:**
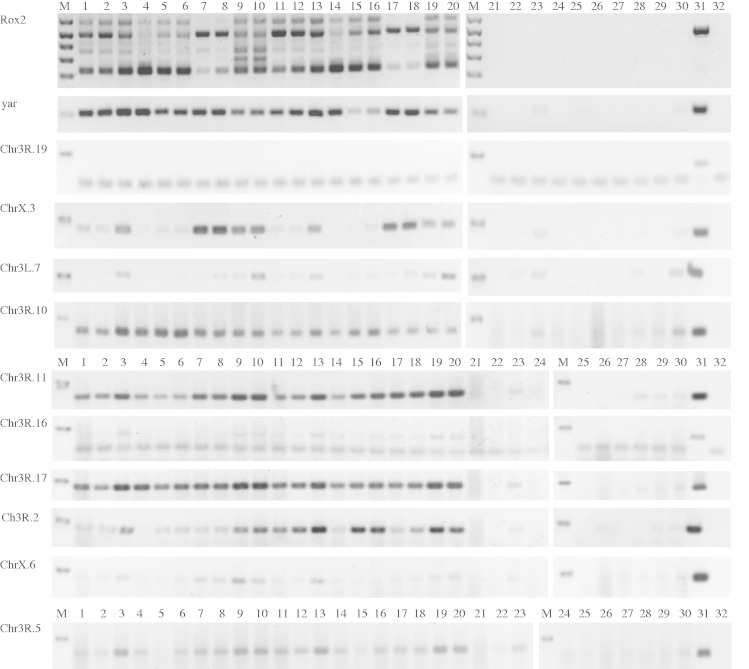
RT-PCR analysis of ultra-conserved elements. M represents DNA size marker, in which *Rox2* shows 100–500 bp bands in 100 bp increments plus a 650 bp band; all other samples show the 100 bp band. Lanes 1–10 are RT-PCRs from cDNA synthesized using oligo-dT, lanes 11–20 are RT-PCRs from cDNA synthesized using random hexamers, 21–30 are RT- controls using random hexamer. Lanes 1, 2, 11, 12, 21, and 22 are RNA extracted from 0 to 4 h embryos; 3, 4, 13, 14, 23, and 24 are from 10 to 20 h embryos; 5, 6, 15, 16, 25, and 26 are from 3 - to 4-day-old larva; 7, 8, 17, 18, 27, and 28 are from 3- to 5-day-old female flies; 9, 10, 19, 20, 29, and 30 are from 3- to 5-day-old male flies; lane 31 is a PCR positive control using genomic DNA as template, lane 32 is PCR negative control using water as template.

We hypothesized that some of these UCEs are transcribed, but at low enough levels not to be detected using hybridization or RNA-Seq. We performed RT-PCR reactions from two biological replicates of five developmental stages for ten intergenic UCEs, using the ncRNA genes *rox2* and *yar* as positive controls (fig. 5). 3R.19 is not expressed, whereas 3L.7 and X.6 show possible low-level expression but the results are ambiguous because RT-minus controls showed some contamination. 3R.16 appears to have low-level expression at all stages. The remaining six show clear evidence of expression. Most are expressed at all stages, although X.3 shows higher expression in adults, particularly males. Most also show equal or greater expression in oligo-dT-primed cDNA, suggesting that their transcripts are polyadenylated (Tupy et al. 2005). We note that among the elements that we found evidence for transcription in modENCODE and hybridization data, all are transcribed in our RT-PCR experiment if one includes the ambiguous results at 3L.7 and X.6.

## Discussion

Determining the complete catalog of functional elements within a genome remains a crucial goal to modern genomics (e.g., Birney et al. 2007; Roy et al. 2010; Dunham et al. 2012). Experimental approaches through the use of large-scale genomic technologies have been successful at capturing many such functional elements (e.g., Dunham et al. 2012). A complimentary approach is to use comparative genomic information that leverages patterns of sequence conservation for the discovery of elements that are maintained by natural selection over long timescales of evolution. The implicit assumption in this evolutionary analysis is that conservation over evolutionary time implies function and such comparative analysis has proven extremely valuable (e.g., Pollard et al. 2006; Stark et al. 2007).

UCEs, those elements that have remained completely unchanged over the course of evolutionary time, must be critically important to organismal fitness, and accordingly studies from human populations have shown that patterns of variation are consistent with the action of extremely strong selection (Katzman et al. 2007; Chiang et al. 2008; McLean and Bejerano 2008). However, we still have little understanding of the function of these genomic elements. Here, we have discovered a set of *Drosophila* UCEs that have been conserved over the course of hundreds of millions of years of evolution. Previously, Glazov et al. (2005) defined a set of UCEs on the basis of a more limited three-way alignment of *D*. *melanogaster, D*. *pseudoobscura,* and *Anopheles gambiae*. Those UCEs provided evidence that fly UCEs might often have conserved RNA secondary structures, particularly as associated with regulatory functions in the genome. Our own set, based on a more complete phylogenetic sampling, provides additional evidence of this trend. Further, our population genetic inference (table 1) suggests that UCE variation experiences strengths of selection that are an order of magnitude stronger than segregating amino acid variation across two populations with very different demographic histories. This is considerably stronger than what has been observed in humans, where mutations at ultraconserved positions were found to be under roughly 3-fold stronger selection than nonsynonymous variation (Katzman et al. 2007). Thus our elements are very likely to be functional.

*Drosophila* UCEs show many of the same features as human UCEs: 1) the majority occur in intergenic regions of the genome, 2) those elements that occur within exonic or intronic regions cluster in genes responsible for crucial early developmental phenotypes, and 3) intergenic elements show distinct patterns of base composition whereby A + T frequency dips in flanking regions of UCEs and then rises in the central regions of the elements.

A subset of mammalian UCEs harbor ncRNAs that when altered can lead to human leukemias and carcinomas (Calin et al. 2007). We have discovered a set of novel ncRNAs associated with *Drosophila* UCEs. These are prime candidates for future experimental studies because our population genomic analyses strongly suggest that these *Drosophila* UCEs are highly constrained and thus functional.

## Supplementary Material

Supplementary figures S1–S4 and tables S1–S4 are available at *Genome Biology and Evolution* online (http://www.gbe.oxfordjournals.org/).

Supplementary Data
