# Supplementary material for: Highly Constrained Intergenic Drosophila Ultraconserved Elements Are Candidate ncRNAs
Source: Genome Biol Evol. 2015 Jan 23;7(3):689–98. doi: 10.1093/gbe/evv011 (PMC5322558; doi:10.1093/gbe/evv011)
Supplement: Supplementary Data [file supp_7_3_689__index.html]

Highly constrained intergenic Drosophila ultraconserved elements are candidate ncRNAs — Highly Constrained Intergenic Drosophila Ultraconserved Elements Are Candidate ncRNAs — Supplementary Data 

# Highly Constrained Intergenic *Drosophila* Ultraconserved Elements Are Candidate ncRNAs

## Supplementary Data

files

**Files in this Data Supplement:**

- Supplementary Data - docx file
- Supplementary Data - xlsx file
