# Supplementary material for: Highly Constrained Intergenic Drosophila Ultraconserved Elements Are Candidate ncRNAs
Source: Genome Biol Evol. 2015 Jan 23;7(3):689–98. doi: 10.1093/gbe/evv011 (PMC5322558; doi:10.1093/gbe/evv011)
Supplement: Supplementary Data [file supp_evv011_flyUltras_suplFigs.docx]

Figure S1. MCMC chain convergence for flanking UCE regions. Shown is the marginal distribution of the posterior likelihood throughout the first 10000 samples of a set of independent chains. The first half of each simulation was treated as burn-in and discarded, the second half was used for estimation.

Figure S2. MCMC chain convergence for nonsynonymous SNPs. Shown is the marginal distribution of the posterior likelihood throughout the first 10000 samples of a set of independent chains. The first half of each simulation was treated as burn-in and discarded, the second half was used for estimation.

 Figure S3. MCMC chain convergence for UCE regions. Shown is the marginal distribution of the posterior likelihood throughout the first 10000 samples of a set of independent chains. The first half of each simulation was treated as burn-in and discarded, the second half was used for estimation.

Figure S4. Predicted secondary structures from 7 UCEs chosen from among our broader set of non-coding UCEs to demonstrate a common stem-loop structure seen in many predicted UCE structures.
